# Anticancer of genus *Syzygium*: a systematic review

**DOI:** 10.37349/etat.2023.00134

**Published:** 2023-04-27

**Authors:** Mahmoud Dogara Abdulrahman, Harmand A. Hama

**Affiliations:** Biology Education Department, Faculty of Education, Tishk International University, Kurdistan Region, Erbil 44001, Iraq; NGO Praeventio, Estonia

**Keywords:** Anticancer, activity, cancer, plants

## Abstract

**Aim:**

One in eight fatalities globally are considered cancer-related. The need for cancer therapy is growing. Natural products continue to play a role in drug development, as up to 50% of authorized drugs in the last 30 years have been isolated from natural sources.

**Methods:**

Anticancer, antioxidant, antibacterial, antifungal, antiviral, analgesic, anti-inflammatory, and other actions have all been reported in research papers using plants from the *Syzygium* genus in the treatment and prevention of disease.

**Results:**

Results from the anticancer test showed that the genus, especially *Syzygium aqueum*, *Syzygium samarangense,* and *Syzygium cumini* had significant promise as an anticancer agent *in vitro* against several cancer cell lines. Numerous factors, including phytochemical composition, increased apoptotic activity, decreased cell proliferation, stopped angiogenesis, and reduced inflammation.

**Conclusions:**

These results, despite preliminary, show promise for further purification and investigation of bioactive compounds and extracts within the genus *Syzygium* for their anticancer properties.

## Introduction

Cancer is a group of diseases defined by the unchecked growth and spread of cells. Cancer is a major killer worldwide. In 2008, cancer was responsible for 7.6 million deaths worldwide, the vast majority of which occurred in low-income regions [1]. This number is expected to keep rising due to demographic shifts and the prevalence of risky lifestyle choices. Despite the widespread scientific evidence, many of us still don’t know that poor dietary habits are a major cause of cancer [2]. Humans are more likely to develop cancer due to the high levels of chemicals, preservatives, and coloring agents in the food consumed regularly [2]. The inability of anticancer medications to discriminate between cancer cells and healthy cells, as well as the resistance of cancer cells to chemotherapeutic agents, are major factors in the failure of cancer treatment [3]. The need for cancer therapies is growing. Consequently, it is crucial and essential to look for novel compounds that can be used to cure various forms of cancer. Natural products continue to play a role in drug development, as up to 50% of authorized drugs in the last 30 years have been isolated from natural sources. Novel sources of bioactive chemicals in medicinal plants with promising anticancer potential. *Syzygium* belongs to the family Myrtaceae, which includes 3,800–5,800 different species and 140 different genera. *Syzygium*, on the other hand, has 1,100–1,200 species and is found primarily in tropical and subtropical regions of the world, making it the biggest woody genus of flowering plants [4]. Some *Syzygium* species are currently enjoying a boom in popularity in domestic and foreign markets in addition to their centuries-long use as spices, food preservers, and medicinal herbs. A wide range of biological activity have been reported in research papers using plants from the *Syzygium* genus in the treatment and prevention of disease [5–8]. In our search for information, we were unable to locate up-to-date analysis of the effectiveness of genus *Syzygium* against cancer. Not many reviews have been done on the genus as a whole with regard to cancer except [9]. Therefore, this systematic review discussed in detail the great potential for obtaining the lead drug from chemical constituents of various species from the genus *Syzygium* as the anticancer.

## Material and methods

### Inclusion criteria

Science Direct, PubMed, Wiley, Springer, Sage, Google Scholar, and Hindawi were among the online resources that mined primary research papers for relevant data. To be considered for inclusion, a piece must meet the following criteria: Our search terms included “anticancer”, “breast”, “colon”, “liver”, “lungs”, “skin”, “stomach”, “cervical”, “*Syzygium*”, “phytochemical”, “chemical”, “crude extract”, “HPLC analysis”, “FTIR analysis”, and “gas chromatography-mass spectrometry (GC-MS) analysis”.

### Exclusion criteria

We did not include information from sketchy websites in our analysis. None of the articles were considered since they were written in a language other than English, and this includes thesis papers and reviews.

## Results

### Taxonomy, origin, and distribution of genus *Syzygium*

Aroma, essential oil, flower composition, plant structure, and phloem distribution are only a few of the characteristics used to categorize members of the Myrtaceae family [10]. In 1893, the Myrtaceae were divided into two groups, the Leptospermoideae and the Myrtoideae, based on whether the plants had opposite or alternate leaves and capsular or fleshy fruits [4]. In 1984, it is argued that the taxonomic classification of the family Myrtaceae should be based on the morphological features of the species within the family, and they validated their arguments with molecular studies, which led them to the conclusion that there are only two families and seventeen tribes: the Psiloxyloideae and the Myrtoideae [11]. The family Myrtaceae has been found to have numerous species and a great deal of genus variety. In the family Myrtaceae, the Syzygieae tribe accounts for most of the species. The genera *Syzygium* and *Psidium*, followed by *Eugenia*, are the most widely planted in the family Myrtaceae. *Syzygium* has 1,200–1,500 species, *Eugenia* has about 1,150, and *Eucalyptus* has around 700 [10]. The complexity and difficulty of taxonomic identification can be traced back to the family’s high level of species diversity. Numerous genera within this family have attracted attention for their potential commercial value since their medical and industrial applications have been recognized the world over. *Syzygium* is a vast family of plants that ranges from southern India and southeast China to southeast Australia and New Zealand [12]. However, whereas Malaysia is the canter of the genus in terms of species richness, it appears that the Malaysian-Australian region is the canter of the genus in terms of its basic evolutionary diversity. Multiple species belong to this genus, which extends from southern East Asia and the Pacific to Africa and Madagascar [13]. They are extremely fragrant plants, and most of the species in this genus are used medicinally. Additionally, the fruits are taken fresh, and their flavour composition has demonstrated that they are also aromatic.

### Traditional uses of some members of the genus *Syzygium*

Chemical, genetic, and molecular diversity are the most prominent types of plant variety, but there is also a great deal of species diversity and eco-climatic adaptations among plants across the globe. Humans from all walks of life and all corners of the globe have long relied on the healing properties of nature’s plants, using them for anything from food to prescription drugs. In many parts of the world, people turn to plants as a complementary method of treating cancer. Those that are less harmful to healthy cells, have less of an adverse biological impact, and have evolved in tandem with their targets are preferred [3]. *Syzygium aqueum* is cultivated for its edible fruits, leaves, and bark. Fever, headaches, gastrointestinal issues, diabetes, high cholesterol, skin problems, and even some forms of cancer can all be alleviated by eating apples [14]. It’s no secret that *Syzygium aromaticum* is a staple in Indian cooking [15]. *Syzygium aromaticum* is utilized as a warming and stimulating stimulant in both Indian and Chinese traditional medicine [16]. *Syzygium aromaticum* essential oil has long been used to treat burns and wounds, as well as a pain reliever in dental care, as well as to cure tooth infections and toothache [17]. It has been utilized by Ayurvedic doctors in India to treat respiratory and digestive disorders since ancient times, and recent scientific research has confirmed its efficacy as a chemo-preventive agent [15]. Sore throat, bronchitis, asthma, thirst, biliousness, diarrhea, and ulcers are only some of the conditions that can be alleviated by using *Syzygium cumini* bark [18]. *Syzygium cumini* leaf juice is administered alone or in combination with carminatives like cardamom and cinnamon to treat diarrhea in children [19], *Syzygium cumini* leaves, mango leaves, and myrobalan leaves are combined with goat’s milk, honey, and carminatives like cardamom and cinnamon to treat dysentery with a bloody discharge [18]. *Syzygium cumini* has historically been used to treat a number of ailments, such as diabetes, inflammation, and diarrhea [20]. *Syzygium jambolanum* fruits are considered spleen disease-treating, tonic, astringent, and carminative [21]. Both pharyngitis and ringworm infections are alleviated with the use of the fruits and seeds of *Syzygium jambolanum* [21]. The fruits of *Syzygium jambolanum* have a sharp, sugary flavor and are also chilly, dry, and astringent to the digestive tract [21]. The plant *Syzygium zeylanicum* has a long history of usage in traditional medicine for conditions like fever, headaches, arthritis, and joint discomfort [22]. Malay people have historically utilized *Syzygium polyanthun* leaves and roots to manage and treat endometriosis, ulcers, hypertension, and diabetes [23]. In Africa, *Syzygium guineense* is used to treat malaria, stomach aches, and ringworm by applying the root, leaf, bark, or fruit [24, 25]. For the creation of evidence-based medications, we give an overview of the traditional applications of a few selected *Syzygium* species. This genus has many species that are utilized in traditional medicine. Analysis of the criticism of these species in light of current knowledge of anticancer activity is crucial because it could eventually close the gap between conventional wisdom and evidence-based research.

### Anticancer

**Figure 1.**
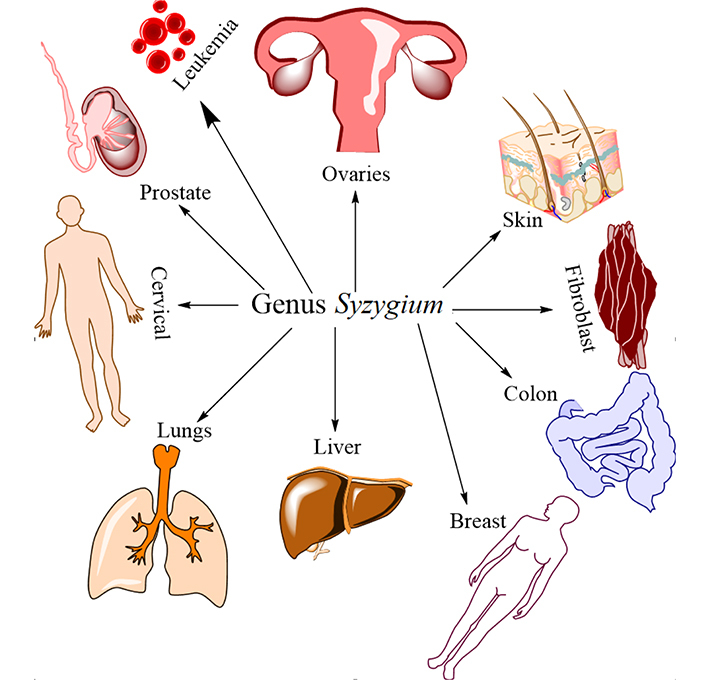
Cancer treated with different members of the genus *Syzygium*

In contrast, extracts from medicinal plants can be used to effectively suppress cancer cell malignancy without causing the harmful effects that come with chemotherapy drugs [26]. There has been a recent uptick in the investigation of medicinal plants for their potential anticancer effects due to the urgent need to discover new, safe, and effective therapeutic agents. The U.S. National Cancer Institute (NCI) states that a half maximal inhibitory concentration (IC_50_) value of less than 20 μg/mL for crude extract and less than 4 μg/mL for purified chemicals is required for anticancer activity [3]. Ideal anticancer drugs target cancer cells alone, killing or crippling them while leaving healthy cells unharmed [27]. Modulating the cell cycle is getting a lot of interest because of the central role it plays in cancer progression. Herbs that have been shown to elicit cell cycle arrest have the potential as both cancer preventatives and therapeutics. Genus *Syzygium* was found to be active against several cancer diseases (Figure 1 and Tables 1–7). Results from *Syzygium aqueum* demonstrated that both substances inhibited the proliferation of cancer cells, with arjunolic acid demonstrating the strongest activity against HeLa cell lines [28]. Fruit extract of *Syzygium aqueum* was found to have antiproliferative activity against MCF-7, a cell line that is highly dependent on the hormone estrogen, suggesting that a component in the extract is responsible for its cancer-fighting abilities. Plant polyphenols have been linked in multiple studies to a reduction in breast cancer metastasis [29].

**Table 1.** Biological evaluation of *Syzygium aqueum* (Burm.f.) Alston against several cancer cell lines

***N***	**Parts of the plant**	**Solvents**	**Concentration**	**Type of cancer**	**Major finding**	**Reference**
1	Stem bark (oleanolic acid and b-sitosterol)	Methanol	100 μL	Cervical, breast	It was found compound one to be significant against the tested cell line while compound 2 was only on HeLa cell line	[30]
2	Leaves (essential oils)	Dimethyl sulphoxide	1%	Breast	As measured by an IC_50_ value of 76.40 μg/mL, it exhibited modest activity against the MCF-7 cell line	[31]
3	Stem bark	Methanol	1.5625–100 μg/mL	Cervical, breast, lung	The IC_50_ values for alphitolic acid were 16.12 mg/mL and 7.37 mg/mL, indicating that it was toxic to the HeLa and T47D cell lines. A549 cells were inhibited to a modest degree (IC_50_ = 84.41 μg/mL), nevertheless. Arjunolic acid was also discovered to be toxic to HeLa cell lines, having an IC_50_ of 6.74 μg/mL	[28]
4	Fruits	Aqueous, methanol	-	Breast cancer cell line	Aqueous extracts revealed antiproliferation effects on MCF-7 cell lines (*P* < 0.05) after 72 h, however, the effects were insignificant on a non-cancer-origin cell line and were not seen in the 24-h or 48-h time periods	[29]
5	Leaves	Methanol	0.01, 0.1, 1, 10, and 100 μg/mL	Breast	At 100 μg/mL, *Syzygium aqueum* was observed to significantly (*P* < 0.05) boost MCF-7 cell growth	[32]
6	Stem bark	Methanol	100 μL	Cervical, breast cancer	Butyrospermol was only moderately cytotoxic (43.59 μg/mL) against the HeLa cell line and barely cytotoxic at all against the T45D and A549 cell lines. And when tested with the 2,3-bis [2-methyloxy-4-nitro-5-sulfophenyl]-2*H*-tetrazolium-5-carboxanilide (XTT) assay, sitosterone (29.96 μg/mL) exhibited considerable toxicity towards the A549 cell line	[33]
7	-	-	50 μg/mL and 100 μg/mL	Fibroblasts, breast	In addition, neither the ethanolic nor the aqueous extracts showed any antiproliferative effects on 4T1 or 3T3 cells at 50 μg/mL or 100 μg/mL	[34]

-: not applicable

**Table 2.** Biological evaluation of *Syzygium samarangense* (Blume) Merr. & L.M.Perry against several cancer cell lines

***N***	**Parts of the plant**	**Solvents**	**Concentration**	**Type of cancer**	**Major finding**	**Reference**
1	Leaves	-	50, 100, 150, and 200 μg/mL	Lung	When tested on A549 cells, the IC_50_ for the green produced silver nanoparticles (AgNPs) was 87.37 μg/mL.	[1]
2	Leaves	Aqueous	100 μg/mL and 250 μg/mL	Skin	In human HepG2-C8 cells with antioxidant response element (ARE)-luciferase plasmids transfected stably, the aqueous extract at 100 μg/mL and 250 μg/mL induced the nuclear factor erythroid 2-related factor 2 (Nrf2)-ARE pathway. Furthermore, the transformation of mouse epidermal JB6 P+ cells was blocked by 12-*O*-tetradecanoylphorbol-13-acetate (TPA) efficiently, suggesting that the extract may have some therapeutic potential.	[35]
3	Fruits	-	100 μL	Lung	There was a notable and concentration-dependent impact on the cell viability of the extracts that were tested. The IC_50_ value indicates that a concentration of 21.86 μg/mL is required to achieve 50% inhibition of proliferation. Ladder-shaped DNA fragments in a DNA fragmentation assay are a biological indicator of intrinsic apoptotic cell death. Morphological alterations in cells treated with the extract confirmed its ability to trigger apoptosis.	[36]
4	Fruit	Methanol	-	Colon	The human colon cancer cell line SW-480 was tested and found to be sensitive to the cytotoxic effects of three C-methylated chalcones (IC_50_ = 10, 35, and 35 μmol/L, respectively). The compounds 2’,4’-dihydroxy-3’,5’-dimethyl-6’-methoxychalcone (1), 2’,4’-dihydroxy-3’-methyl-6’-methoxychalcone (stercurensin, 2), and 2’,4’-dihydroxy-6’-methoxychalcone (cardamonin, 3) all belong to the family of chalcones.	[37]
5	Leaves	Methanol	50, 5, and 0.5 μg/mL	Ovarian	The MCF-7 cell line was shown to be particularly sensitive to the cytotoxic effects of 20,40-dihydroxy-60-methoxy-30,50-dimethylchalcone (IC_50_ = 0.0015 nmol/L). Against the SKBR-3 cell line, it was cytotoxic with an IC_50_ of 0.0128 nmol/L.	[38]
6	Leaves	Ethanol	-	Liver, breast	Both the HepG2 and MDA-MB-231 cells examined responded favorably to the isolated compounds. With IC_50_ values between 1.73 μmol/L and 32.0 μmol/L for HepG2 cells and between 4 μmol/L and 37 μmol/L for MDA-MB-231 cells, all substances examined showed strong cytotoxic effects.	[39]
7	Leaves	50% ethanol	100 μL	-	For inhibiting inhibition of poly(ADP-ribose) polymerase-1 (PARP-1), the IC_50_ for pure vescalagin was 2.67 μmol/L, and for castalagin it was 0.86 μmol/L.	[40]
8	Leaves	Ethanol, acetone, petroleum ether	-	Cervical	The IC_50_ value for the extract was 40.5 μg/mL when tested on the HeLa cell line.	[41]

-: not applicable

**Table 3.** Biological evaluation of *Syzygium aromaticum* (L.) Merr. & L.M.Perry against several cancer cell lines

***N***	**Parts of the plant**	**Solvents**	**Concentration**	**Type of cancer**	**Major finding**	**Reference**
1	Unopened flower bud	Aqueous	100 μL/mouse per day from the fifth week	Lung	In these BP-induced lung lesions, clove therapy significantly decreased the number of proliferative cells and increased the number of apoptotic cells.	[42]
2	Bud	Aqueous, ethanol, essential oil	-	Breast	Essential oil had a median lethal dose (LD_50_) of 37.36 μg/mL and 36.43 μg/mL in the 24-h brine shrimp lethality test (BSLT) and 3-(4,5-dimethylthiazol-2-yl)-2,5-diphenyl tetrazolium bromide (MTT) assays, respectively.	[2]
3	Bud	Aqueous, ethanol, essential oil	-	Cervical, breast, prostate, esophageal	Within 24 h, oil at 300 μL/mL induced 80% cell death in an esophageal cancer cell line through apoptotic cell death, while prostate cancer cells displayed negligible cell death.	[15]
4	Leaves, stem, bark	Methanol	-	Breast	The stem extract exhibits a strong and focused cytotoxic impact on MCF-7 cells, with an IC_50_ of 33 μg/mL.	[43]
5	Eugenol	-	0.1, 0.2, 0.5, 1, 2, 4, and 8 ng/mL	Cervical	Apoptosis was detected by the IC_50_ at 81.85% cell viability.	[44]
6	Eugenol	-	1,200, 600, 300, 150, and 75 μg/mL	Breast, skin	The IC_50_s of chitosan nanoparticles containing *Syzygium aromaticum* essential oil (SAEO) and eugenol against melanoma (A-375) cells were 73 μg/mL and 79 μg/mL, respectively; for breast (MDA-MB-468) cells, the values were 177 μg/mL and 51 μg/mL.	[45]
7	Flower buds	Chloroform	-	Lung	Compared to the control group, the extract fractions inhibited wound closure/cell migration in A549 and H1299 and caused apoptosis in H1299. Nuclei of fraction-treated cells showed signs of apoptosis, including chromatin compression, nuclear shrinkage, and the development of apoptotic bodies.	[46]
8	-	-	2, 3, and 4 mg/mL	Cervical	The extract was tested on HeLa cells and found to have an LD_50_ of 2 mg/mL after being exposed to the cells for 24 h. In comparison to untreated control cells, treated cells were rounded off with a distinctive death symptom.	[47]
9	Eugenol	-	0, 50, 100, and 200 μmol/L	-	Exhibited cytotoxicity in the HeLa cell line at concentrations between 50 μmol/L and 200 μmol/L.	[48]
10	-	Ethanol	1.25, 12.5, 50, 75, and 100 μg/mL	-	When cancer cells were treated with fluorescent magnetic submicronic polymer (FMSP)-nanoparticles alone, their viability dropped to 55.40%, and when crude clove extracts were additionally added to the treatment, viability dropped to 8.50%.	[49]
11	Bud	-	125, 62.5, 32, and 15 μg/mL	Colon	The IC_50_ value for *Syzygium aromaticum* bud essential oil nanoemulsion (SABE-NE) after 48 h was determined to be 74.8 μg/mL.	[50]
12	Bud	Ethanol	0–1,000 μg/mL	Breast	The IC_50_ values for the extract and nanoparticles were determined to be 20 μg/mL and 7 μg/mL, respectively, in an *in vitro* assay.	[51]
13	-	Essential oil	0, 30, 60, and 120 μg/mL	Cervical	By significantly decreasing HeLa cell viability at 24-h and 48-h at *P*-value < 0.0001, the essential oils demonstrated substantial antiproliferative activity. By 48 h, almost no cancer cells had survived at the maximal dose of 120 μg/mL, which not only inhibited proliferation but also drastically reduced the number of HeLa cells.	[52]
14	Buds	Aqueous	10–100 mg/mL	Lung, breast	Phyto-mediated AgNPs had an IC_50_ of 60 μg/mL against MCF-7 and 50 μg/mL against A549 cells. The extract having IC_50_ was also found to be 70 μg/mL against MCF-7 and 70 μg/mL against A549 cells.	[53]
15	Essential oil	-	1.25–20%	Breast, leukemia, cervical	When tested on Hela, MCF-7, and K-562 cell lines, 20% essential oil showed the highest percentage of inhibition, 32.8%, 53.5%, and 76.4%, respectively.	[54]
16	Buds	Methanol, aqueous	-	Breast, colon, liver	According to the data, the IC_50_ values were as follows: 31 μg/mL for colon cancer protection against breast cancer, 29.7 μg/mL and 18.7 μg/mL against liver cancer	[55]
17	Bud	70% ethanol	-	Ovarian	Substantial inhibitory effect against human ovarian cancer cells (A2780; IC_50_ value = 22.67 μmol/L).	[56]
18	Buds	Acetonic, dichloromethane, ethanolic, petroleum ether	-	Colon carcinoma	The ethanolic extract of clove was the most effective against the HCT cell line, with an IC_50_ of 2.53 μg/mL.	[26]
19	Essential oil	-	0.39, 0.781, 1.562, 3.125, 6.25, 12.5, 25, 50, 100, and 200 mg/mL	Colon	At 3.25 mg/mL of SAEO, the maximum cell vitality was recorded, whereas increasing the concentration of the essential oil to 200 mg/mL resulted in relatively low cell viability.	[57]
20	Buds	Methanol	-	Cervix, breast, lung	When cell death is tested on HeLa cells, the median lethal concentration (LC_50_) was 88 ± 3.4 μg/mL, whereas on MCF-7 cells, it was 86 ± 2.8 μg/mL.	[58]
21	Leaves, buds, flower	50% ethanol	6.25, 12.5, 25, 50, 100, 200, and 400 μg/mL	Breast	The IC_50_ values for cytotoxicity against Hela and MDA-MB-231 cell lines were 40 μg/mL and 48 μg/mL, whereas those for flower buds and young flower buds were 35 μg/mL and 39 μg/mL.	[59]
22	-	-	-	-	The relative IC_50_ value of the ethanolic clove extract was 6.8 μg/mL, making it the most potent antiproliferative agent tested.	[60]
23	-	-	-	Breast	The accumulation of nanoparticles in the sub-G1 phase of the cell cycle after treatment with extract coated with polyvinylpyrrolidone (PVP) iron oxide nanoparticles and PVP iron oxide nanoparticles in MCF-7 cell lines confirmed the induction of apoptosis.	[61]
24	Flower buds	Ethanolic	-	Cervical	Cell viability was found to decrease after treatment with the extract in a dose- and time-dependent manner, suggesting an antiproliferative action (*P* < 0.05).	[62]
25	Flower	Aqueous	50–1,000 ppm	-	LC_50_ values of 227.1 g/mL showed that the extract is toxic to larvae.	[63]

-: not applicable

**Table 4.** Biological evaluation of *Syzygium cumini* (L.) skeels against several cancer cell lines

***N***	**Parts of the plant**	**Solvents**	**Concentration**	**Type of cancer**	**Major finding**	**Reference**
1	Partially ripe fruit skin	Methanol	50 μL of 100% or 10% extract	Cervical	Inhibition of growth was seen at 14.4% (HeLa) and 11.8% (SiHa) at a concentration of 40%, and at 30.3% and 23.2%, respectively, at a concentration of 80% of the extract.	[64]
2	-	-	-	Colorectal	Extract was found to significantly inhibit the proliferation of HT-29 cell lines. After treatment, there was also a notable shift in the intended gene expression ratio (*Bax*:*Bcl*-*2*).	[65]
3	Seeds	-	25 mg/kg body weight (b.wt). per day	Stomach	Groups significantly increased phase II detoxifying enzymes and inhibited lipid per oxidation in the stomach, leading to a decrease in tumor incidence, tumor burden, and cumulative number of gastric carcinomas.	[66]
4	Fruits	Chloroform	0, 0.5, 1.0, 2.5, and 5 μg/mL	Ovary	More than 90% cell cytotoxicity was seen with quercetin and gallic acid at concentrations of 2.5 μg/mL and higher, but oleanolic acid was only modestly effective up to 5 μg/mL in a serial dilution.	[67]
5	Leaves	Ethyl acetate, methanol, aqueous	31.25, 62.25, 125, 250, 500, and 1,000 μg/mL	Cervical	The IC_50_ values for ethyl acetate (350 μg/mL), methanol (378 μg/mL), and aqueous (360 μg/mL), respectively, ranged from moderately toxic to non-toxic (no effect).	[3]
6	Leaves, pulp, and seeds	Ethanol	100 μL	Breast	Seeds, leaves, and pulps all had IC_50_ values of 613, 660, and 732 μg/mL, respectively.	[68]
7	Pulp, seeds	Ethanol	12.5–200 μg/mL	Lung	Hydrolysate pulp exhibited potent antiproliferative action with an IC_50_ value of 59 μg/mL ± 4 μg/mL. The hydrolyzed seed extract was the most effective on cell proliferation (IC_50_ = 38 μg/mL ± 3 μg/mL at *P* < 0.05).	[69]
8	-	Methanol	10, 20, and 40 μg/mL	Oral	The oral squamous cell carcinoma (OSCC) cell line was cytotoxic after the treatment, and intracellular reactive oxygen species (ROS) buildup was generated. This therapy also triggered apoptosis-related morphological alterations and the extirpation of phosphatidylserine in OSCC cells. Protein and gene expression of cadherin-1 were also boosted by the treatments.	[70]
9	Seeds	Ethanol	-	Ovary, lung	The IC_50_ value for the A2780 (ovarian cancer) cell line was 49 μg/mL, whereas that for the H460 (non-small cell lung carcinoma) cell line was 165 μg/mL. The IC_50_ values for flavopiridol (positive control) ranged from 0.06 μg/mL to 0.08 μg/mL across all cell lines.	[71]
10	Seeds	Ethanol	125 mg/kg b.wt. per day for each animal	Skin	Compared to the carcinogen control group, the average latency duration was likewise significantly lengthened in the extract treatment group (pre-group—11.1 weeks; post group—10.9 weeks).	[72]
11	-	Hexane, chloroform, ether, ethyl acetate, ethanol, aqueous	-	Leukemia	Based on the data, the ethanolic extract inhibits human acute myeloid leukemia (AML) cells at a lower concentration than the other extracts (LC_50_ = 81 μg/mL) but at a higher concentration than the pure compounds [b-sitosterol (LC_50_ = 55.0 g/mL), and kaempferol 7-*O*-methylether (LC_50_ = 48.0 μg/mL)], with 91.71% and 100% inhibition, respectively.	[73]
12	Bark	Methanolic	25, 50, and 75 mg/kg per day	Ehrlich ascites carcinoma (EAC)	Extract significantly inhibited EAC cell proliferation (71.08% ± 3.53%; *P* < 0.001), reduced tumor burden (69.50%; *P* < 0.01) and increased the life duration (73.13%; *P* < 0.001) of EAC-bearing mice at 75 mg/kg per day.	[74]
13	Leaves	Ethanol	-	Breast	T47D breast cancer cell line showed the highest cytotoxic activity, with 69% growth inhibition being the best result.	[75]
14	Seeds	Aqueous	250 mg/kg b.wt. per day	Skin	When compared to the carcinogen-treated control group, whose tumor incidence was determined to be 100%, the mice showed a considerable drop to 37.5%, 50%, and 25%, respectively.	[76]
15	Seeds	Methanol	10, 20, and 40 μg/mL	Hepatocellular carcinoma	The cytotoxicity of HepG2 cells was significantly increased by treatment with the extract, and this increase was concentration dependent.	[77]
16	Fruits	-	-	Breast	Significant reductions in tumor incidence (65%), tumor load (313 mm^3^), and tumor multiplicity (1.8 tumors/rat) compared to controls. This treatment also considerably delayed the initial tumor emergence by 21 days.	[78]
17	Unripe fruit pulp	Ethanol	-	Colorectal adenocarcinoma	When evaluated against colorectal adenocarcinoma (Caco2), the methanolic extract of unripe fruit seeds demonstrated the highest anticancer activity at the highest concentration (1,000 μg/mL), with an IC_50_ value of 30.93.	[79]
18	Seeds	Methanol	-	-	50 mg/kg (i.p.) reduced 67.36% (*P* < 0.01) of cell proliferation in EAC cells on day six of incubation.	[80]
19	Fruits	Methanol, aqueous	-	Lung	The extract, at a concentration of 2 mg/mL.	[81]

-: not applicable; *Bcl*-*2*: B-cell lymphoma 2; *Bax*: *Bcl*-*2*-associated X protein

**Table 5.** Biological evaluation of *Syzygium polyanthum* (Wight) Walp. against several cancer cell lines

***N***	**Parts of the plant**	**Solvents**	**Concentration**	**Type of cancer**	**Major finding**	**Reference**
1	Leaves	Methanol	15.63–1,000 μg/mL	Breast	With IC_50_ values of 672 μg/mL and 126 μg/mL against 4T1 and MCF-7 cells, respectively, the extract displays a weak cytotoxic impact.	[82]
2	Leaves	Aqueous	-	-	The cell cycle arrest of HB4C5 is induced by the crude extract between G1 and S phase.	[83]
3	Ripened and unripe fruits, leaves	Ethanol	-	-	Fruit and leaf extracts were found to be ineffective (LC_50_ > 1,000 μg/mL).	[84]

-: not applicable

**Table 6.** Biological evaluation of *Syzygium guineense DC*. against several cancer cell lines

***N***	**Parts of the plant**	**Solvents**	**Concentration**	**Type of cancer**	**Major finding**	**Reference**
1	Leaves, roots, bark	Methanol, ethanol	-	Breast, colon	Strong action against these cancer cells and colon cancer (CC) organoids was shown by the extract. Extraction effects on triple-negative breast cancer (TNBC) cell proliferation corresponded to suppression of the Wnt3a-induced catenin stabilization and transcriptional response	[85]
2	Stem	Aqueous	1,000 mg/kg	Liver, colon, skin	50% inhibition of growth (GI_50_) of 50 μg/mL was found to be an effective inhibitor of melanoma cell proliferation.	[86]
3	-	-	-	-	Within the first day, the IC_50_ was 0.0008549 mg/mL. These plant extracts have antiproliferative capabilities that could be investigated	[87]

-: not applicable

**Table 7.** Biological evaluation of some members of genus *Syzygium* against several cancer cell lines

***N***	**Species name**	**Parts of the plant**	**Solvents**	**Concentration**	**Type of cancer**	**Major finding**	**Reference**
1	*Syzygium alternifolium* (Wight) Walp.	Leaves	Hexane, methanol	10, 25, 50, and 100 μg/mL	Breast, prostate	The human cancer cell lines MCF-7 and DU-145 had IC_50_ values of 8.177 μg/mL and 2.687 μg/mL for leaf hexane extract, respectively.	[88]
2	*Syzygium calophyllifolium* (Wight) Walp.	Bark	Methanol	5, 10, and 20 μg	Breast	When compared to the control, MCF-7 cells cultured with SCBM extract for 24 h lost their original shape at increasing concentrations. Membrane damage, cell rounding, and cell separation from the culture plates were all telltale markers of cell death. At the smallest dose, however, these effects were not observed.	[89]
		Leaves	Ethyl acetate	1:1 to 1:64	Monolayer culture	As sample concentration increases, cell viability declines. When the extract was concentrated, further, 88.53% of the cell lines died.	[12]
3	*Syzygium anisatum* (Vickery) Craven & Biffin	-	-	0–2.0 mg/mL	Glandular, fibroblast, bladder, liver	Caused a 25%–50% death rate in HepG2 hepatocellular cancer cells. Colon cancer cells (HT-29; IC_50_ = 0.75–1.39 mg/mL), stomach cancer cells (AGS; IC_50_ = 0.59–1.88 mg/mL), bladder cancer cells (BL13; IC_50_ = 0.56–1.12 mg/mL), and liver cancer cells (HepG2; IC_50_ = 0.38–1.36 mg/mL) all had their proliferation inhibited by the extracts. Non-transformed colon cells (CCD-18Co; IC_50_ > 2.0 mg/mL) and stomach/intestine cells (Hs 738. St/Int; IC_50_ > 2.0 mg/mL) showed no discernible loss of viability.	[90]
4	*Syzygium austral* (J.C.Wendl. ex Link) B.Hyland	Fruit	Methanol, aqueous, ethyl acetate	30 μL	Colon, cervical	Against CaCo2 and HeLa cells, the aqueous extracts showed the greatest activity, with IC_50_ values of 27 μg/mL and 172 μg/mL.	[91]
5	*Syzygium myrtifolium* Walp.	Leaves	Essential oil	6.25, 12.5, 50, and 100 μg/mL	Colorectal, ovary	The IC_50_ values for the essential oil were 59.9 μg/mL and 47.5 μg/mL for the HCT-116 and human ovarian teratocarcinoma cells (Pa-1) cell lines, respectively.	[13]
6	*Syzygium jambos* (L.) Alston	Leaves	Methanol	15 μg/mL (pure compound), 25 μg/mL	Liver	The research confirmed the extract acted on a cellular level positively affecting the apoptotic cell cycle pathway via *Bcl*-*2* and *Bax* gene expression.	[92]
7	*Syzygium paniculatum* DC.	Fruits	Ethanol	100, 200, and 400 μg/mL	Pancreatic cancer cells (MiaPaCa-2)	Compared to the chemotherapy drug gemcitabine, the extract (200 μg/mL) dramatically decreased the vitality of MiaPaCa-2 and ASPC-1 pancreatic cancer cells.	[93]
8	*Syzygium malaccense* (L.) Merr. & L.M.Perry	Fruits	Methanol, aqueous	-	Lung, kidney	In the doses used, the extract’s effects on the two cell lines were not statistically significant.	[81]
9	*Syzygium mundagam* (Bourd.) Chithra	Bark	Methanol	-	Breast	Reduced ATP levels (47.96%) and elevated lactate dehydrogenase (LDH) levels (40.96%) in MCF-7 cells were indicative of solitary metachronous bone metastasis (SMBM)-induced toxicity.	[94]
10	*Syzygium zeylanicum* (L.) DC.	Leaves	Methanol	-	-	Both 1:250 and 1:125 had the highest cell viability rates.	[95]
11	*Syzygium coriaceum* Bosser & J.Guého	Leaves	Aqueous methanol (80%, v/v)	-	-	And at 40 μg/mL, *Syzygium coriaceum* caused an 88.1% (*P* < 0.0001) decrease in mitochondrial membrane potential, a 5.7% (*P* < 0.0001) increase in the number of the cell population in G0/G1, and an increased (*P* < 0.0001) proportion of cells experiencing apoptotic/necrotic cell death.	[96]
		Leaves	Aqueous methanol (80%, v/v)	10 μg/mL and 100 μg/mL	Lung carcinoma, liposarm hepatocellular carcinoma	Dose-dependent elevation of ROS was observed after extract treatment in HepG2 cells, with a 4.4-fold rise at 100 mg/mL (*P* < 0.0001). The dose-dependent reduction in antioxidant enzyme activity mirrored the increase in ROS concentration. At 40 μg/mL (*P* < 0.0001), glutathione peroxidase activity dropped by 80.5%.	[97]

-: not applicable

The aqueous extract of *Syzygium samarangense* at 100 μg/mL and 250 μg/mL stimulated the Nrf2-ARE pathway in human HepG2-C8 cells that had been transfected with stable ARE-luciferase plasmids. Additionally, TPA effectively prevented the transformation of mouse epidermal JB6 P+ cells, indicating that the extract may have some therapeutic potential (Table 2). Furthermore, according to a reported study [36], the extracts had an impact on cell viability that was both noticeable and concentration-dependent. A biological sign of intrinsic apoptotic cell death is ladder-shaped DNA fragments in a DNA fragmentation experiment. The extract’s ability to cause apoptosis was demonstrated by morphological changes in cells that were treated with it. It is investigated that the human colon cancer cell line SW-480, and it was discovered that it was susceptible to their cytotoxic effects (IC_50_ values of 10, 35, and 35 μmol/L, respectively) [37]. The family of chalcones includes the substances 2’,4’-dihydroxy-3’,5’-dimethyl-6’-methoxychalcone (1), 2’,4’-dihydroxy-3’-methyl-6’-methoxychalcone (stercurensin, 2), and 2’,4’-dihydroxy-6’-methoxychalcone (cardamonin, 3). It is also concluded that when the *Syzygium samarangense* leaf extract was evaluated using the HeLa cell line, the IC_50_ result was 40.5 μg/mL [41].

*Syzygium aromaticum* essential oil had the greatest cytotoxic effect in both the BSLT and MTT assays, followed by the ethanol and water extracts [2]. Essential oil in the 24-h BSLT had an LD_50_ of 37 μg/mL. In addition, the essential oil’s IC_50_ values in 24 h of MTT assays were 36.43 μg/mL [2]. Oil extract of *Syzygium aromaticum* demonstrated the most cytotoxic activity out of the three types of extracts tested against five distinct cancer cell lines [15]. Morphological examination and 4’,6-diamidino-2-phenylindole dihydrochloride (DAPI) staining confirmed that cell disintegration and subsequent membrane rupture were the cause of cytotoxicity. Within 24 h, clove oil at 300 μL/mL caused the most cell death and apoptotic cell death in esophageal cancer cells [15]. Eugenol, which was found in *Syzygium aromaticum*, may induce apoptosis in order to kill certain cancer cells by exerting its cytotoxic effects [15]. The stem extract has a significant and selective cytotoxic effect on MCF-7 cells, with an IC_50_ of 331.6 μg/mL [43]. The stem extract’s cytotoxic effect was caused by the stimulation of cell death’s apoptotic mechanism [43]. The eugenol isolated from *Syzygium aromaticum* was extremely inhibitive, with effects on cell viability that depended on time and dose as well as consistent morphological changes [44]. Apoptosis was detected by the IC_50_ at 81.85% cell viability (Table 3) [44]. There was evidence of eugenol’s activity in several other cancer cell lines, not just HeLa cells. Regarding promoting cell death, eugenol’s action depends not only on the concentration but also on the dosage and length of exposure. The cell viability of the A-375 cancer cell line was decreased by at least 50% in the presence of both eugenol chitosan-based nanoparticles (EugChiNPs) and *Syzugium aromaticum* chitosan-based nanoparticles (SAChiNPs) at all concentrations examined [45]. Neither eugenol nor SAEO demonstrated any discernible cytotoxicity compared to the control group at doses of 150 μg/mL. Cell viability decreased to under 50% when eugenol and SAEO concentrations (> 600 μg/mL) were increased [45]. The powerful antioxidant, antiproliferative, and antibacterial effects of *Syzygium aromaticum* (clove) are attributed to its tannins, flavonol glycosides, and volatile phenolic oils (eugenol, acetyl eugenol). It is perfect for use as a cancer chemo preventive drug since it has qualities that make it antibacterial, antiseptic, and anti-inflammatory [50]. Eugenol may have an apoptotic effect by reducing cyclooxygenase-2 (COX-2), B-cell lymphoma, and interleukin-1 beta production, and by increasing the activity of caspase-3 and caspase-9 caspase proteins. Therefore, our results suggest that the natural compounds present in *Syzygium aromaticum*, especially eugenol, could be exploited to create a new treatment for esophageal, breast, and cervical cancer (Table 3).

*Syzygium cumini* extracts at 40% concentration inhibited HeLa and SiHa cell growth by 14.4% and 11.8%, respectively; at 80% concentration, the extract inhibited growth by 30.3% and 23.2% [64]. Growth of HT-29 cell lines was significantly inhibited by the *Syzygium cumini* extract [65]. After treatment, there was also a notable shift in the intended gene expression ratio (*Bax*:*Bcl-2*). The results of the DNA damage assay and the apoptotic process suggested by the healing of the wounds indicate that the likelihood of metastasis has decreased following treatment with *Syzygium cumini* extract [65]. Animals in groups V–VII given *Syzygium cumini* extract had higher levels of the non-enzymatic antioxidant protein glutathione (GSH) in their stomachs compared to controls given the carcinogen [66]. *Syzygium cumini* has been found to contain fatty oils, phytosterols, and phenolic compounds. Given the results of the current investigation, it is possible that all of these factors work together to endow this plant extract with its anti-cancer effects [66]. Gallic acid and quercetin extracted from *Syzygium cumini* fruits exhibit > 90% cytotoxic effects at modest concentrations [67]. Based on the results, the IC_50_ of ethyl acetate, methanol, aqueous, and cisplatin (standard) was 330 μg/mL (moderately toxic), 378 μg/mL (moderately toxic), 360 μg/mL (not toxic), and 8.11 μg/mL (very toxic), respectively [3]. From these numbers, it’s clear that the IC_50_ value for the ethyl acetate extract is the lowest. The more toxicity there is, the lower the IC_50_ value. Treatment with *Syzygium cumini* extract (SCE) significantly reduced the number of papillomas present (Table 4). In this study, rats given an oral dose of *Syzygium cumini* seed extract showed no signs of tumor growth over the course of the experiment [72]. While the ethanolic extract’s LC_50_ = 81 μg/mL of anticancer activity was higher than that of the other extract’s (70.7%), it was still lower than the 91.71% and 100% inhibition seen with the pure compounds [b-sitosterol (LC_50_ = 55.0 μg/mL and kaempferol 7-*O*-methylether, LC_50_ = 48.0 μg/mL)] [73]. These results may be explained by the fact that many active components present in the crude extract of ethanol compete. Lung cancer cell viability was found to be considerably decreased by an extract of *Syzygium cumini* at the highest dose (2 mg/mL) [81]. This type of cancer is now thought to be the most frequent because of its large prevalence (millions of new cases annually) [81].

It is reported that a flavonoid fraction isolated from *Syzygium polyanthum* leaves is cytotoxic to mouse colon 26 adenocarcinoma cells and human hybridoma HB4C5 mice [83]. The extract exhibited a modest cytotoxic effect on 4T1 and MCF-7 cells, with IC_50_ values of 672 μg/mL and 126 μg/mL, respectively (Table 5). The active flavonoid component of *Syzygium polyanthum* stacked the cell cycle at G2/M phase, indicating that the effect of cell growth inhibition was not attributable to apoptosis [83].

When the extracts of *Syzygium guineense* were used at a concentration of 50 μg/mL, they fully inhibited the Wnt-dependent TopFlash transcription but had no effect on the constitutive CMV-Renilla transcription [85]. Thus, the active compound(s) from *Syzygium guineense* specifically block Wnt3a-induced β-catenin stabilization transcription in TNBC cells but have no effect on transcription in general [85]. GI_50_ = 50 μg/mL was found to be an effective inhibitor of melanoma cell proliferation from stem extract [86].

After 24 h of treatment, the IC_50_ values for HCT 116 and Pa-1 cell lines were 59.9 μg/mL and 47.5 μg/mL, respectively, indicating strong cytotoxic effects of the leaf essential oil of *Syzygium myrtifolium* compared to control. A higher dose of the essential oil greatly suppressed the expansion of HCT 116 and Pa-1 cell lines [13]. Essential leaf oil’s cytotoxicity may result from the major and minor constituents’ synergistic and cumulative actions of germacrene D, caryophyllene oxide, and caryophyllene.

Cell viability was reduced by 10% and 22% in MiaPaCa-2 and ASPC-1 pancreatic cancer cells, respectively, when treated with *Syzygium paniculatum* extract at 100 μg/mL, but human pancreatic ductal epithelial (HPDE) cells had no effect [93]. Conversely, MiaPaCa-2 and ASPC-1 cell viability were significantly decreased by 77% and 34%, respectively, after incubation with a higher dose of the extract (200 μg/mL, *P* = 0.0004 and *P* = 0.02, respectively). The results were compared to those achieved with gemcitabine, a chemotherapy drug often used as the initial line of defense against pancreatic cancer [93].

Cells treated with *Syzygium mundagam* bark extract were clearly smaller and had more nuclear damage in micrographs. This morphology of cell death was confirmed by Hoechst staining. Reduced ATP levels (47.96%) and elevated LDH levels (40.96%) in MCF-7 cells were indicative of SMBM-induced toxicity [94]. Shape loss, condensed nuclei, distributed nuclear granules, and shattered nuclei may have all contributed to the extract’s potency. Apoptosis may explain the extract’s observed nuclear damage [94]. There is an undeniable demand for less harmful and invasive treatment options for many cancer types. It is indicated that the natural cures *Syzygium aromaticum*, *Syzygium aqueum*, *Syzygium samarangense*, *Syzygium cumini*, and possibly all members of the genus *Syzygium*, inhibit the development of cancer cells by apoptosis and other processes (Table 7). Apparently, they have chemicals that may fight cancer. Some of the ways the compounds or extract prevent cancer progression include increased apoptotic activity, decreased cell proliferation, stopped angiogenesis, and reduced inflammation. The resveratrol molecule found in the *Syzygium* genus can reduce the number of terminal end buds. Resveratrol can also inhibit 5-lipoxygenase (5-LOX) and COX-2 activity.

### Compounds responsible for anticancer in genus *Syzygium*

**Figure 2.**
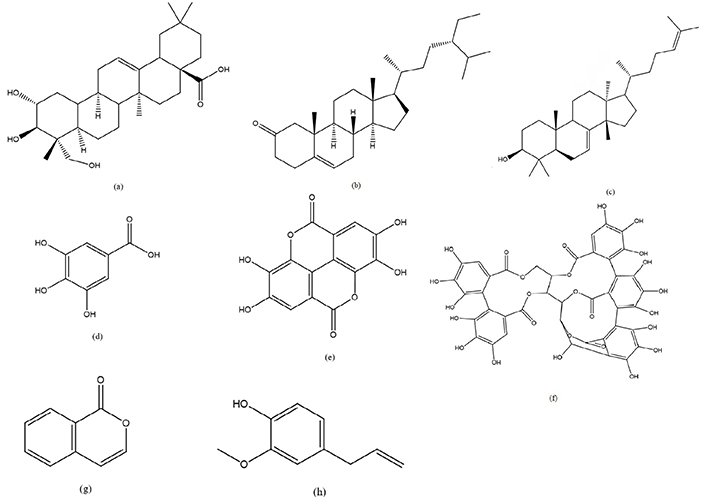
Compounds responsible for anticancer activity in genus *Syzygium*. (a) Arjunolic acid; (b) sitosterone; (c) butyrospermol; (d) gallic acid; (e) ellagic acid; (f) vescalagin; (g) iso coumarin; (h) eugenol

Plants have evolved over many millions of years to create a wide variety of compounds. Each of the several plant chemical groups from which phytochemicals are derived has its own special set of health advantages. Phytochemicals may in some cases shield people from a variety of ailments. Phytochemicals are plant compounds with anti-inflammatory or antioxidant activity but no nutritional value. Although plants create these phytochemicals as a form of defense, it is revealed that they also offer protection against human disease. There are many different phytochemicals found in plants, each with its own unique properties. Polyphenols, a large and varied chemical class consisting of diverse combinations and polymers of A, has been found to have powerful anticancer capacity and may even have protective benefits on human health. A variety of phytochemicals, including flavonoids, phenolics, and polyphenolic, are produced by plants; these compounds are powerful antioxidants and can reduce the negative effects of oxidative stress [74]. Numerous plant-based chemicals have been touted for their purported anticancer, anti-inflammatory, and antioxidant properties. Compounds found in plants have been shown to increase the absorption of a medicine used to prevent cancer cell growth in the digestive tract. The abundance of bioactive phytochemicals in plants like *Syzygium* species makes them a typical base of supplementary treatments. *Syzygium* species has been linked to numerous natural chemicals with anticancer properties, including phenolics, oleanolic acid, betulinic acid, and dimethyl cardamonins (Figure 2). Terpenoids, chalcones, (-)-epigallocatechin, samarangensis A and B, pinocembrin, samarone A–D, jasmonic acid, lignans, alkyl phloroglucinols, hydrolyzable tannins, and other derivatives are all metabolites widely generated by plants in the *Syzygium* genus (Figure 2). The bioactive components in fruits with strong antioxidant and anticancer activities are often polyphenols and their secondary metabolites like flavonoids and proanthocyanidins [93]. Some members of the *Syzygium* genus contain phytochemicals such as sesquiterpenes, monoterpenes, hydrocarbons, phenolic compounds, eugenol, and caryophyllene [63]. Eugenol inhibits the growth of colon, stomach, breast, prostate, melanoma, and leukemia cancers, whereas caryophyllene inhibits the growth of pancreatic, cutaneous, lymphatic, and cervical cancer [98]. Also, it is suggested that eugenol’s anticancer effect was achieved by a combination of mechanisms, including induction of apoptosis, cell cycle arrest, and reduction of proliferation, migration, angiogenesis, and metastasis in a variety of cancer cell lines [63]. Concentration-dependently, arjunolic acid decreased the wound closure rate of PANC-1 cancer cells and triggered a cell-cycle arrest at G0/G1 [99]. Gallic acid controls cancer development and progression by modulating the expression of a number of genes involved in cell death and proliferation [100]. Pathways indicate the probable mechanisms of action that cause G2/M arrest and death in a wide range of cancer cells (Figure 2). G2/M arrest is caused by lowering cell division cycle protein 2 (cdc2), cdc25c, and cyclin B1 and boosting p21^WAF1/CIP1^ [101]. Furthermore, it causes apoptosis by raising the *Bax*:B-cell lymphoma-extra-large (*Bcl-xL*) ratio, caspase 3 activity, and cleaved PARP while lowering pro-caspase proteins (caspase-3, -6, -8, and -9) [101]. In conclusion, there is great potential for the *Syzygium* genus to be developed as an anticancer agent in the future. Through this study, the genus’s latent capabilities have been revealed. Considering these encouraging findings, the potential for a natural substance to play a central role in the next generation of cancer treatments is undeniable. To effectively treat cancer cells, we propose more studies with multidisciplinary directions.

## Discussion

Drug discovery and the development of therapeutic medicines owe a great deal to natural goods, particularly medicinal plants. Many secondary metabolites with various biological or pharmacological effects can be found in the genus *Syzygium*. The information was collected and analysed from the research articles. Revealed members of the genus *Syzygium* are traditionally used in the treatment of cancer and based on the *in vitro* and few *in vivo* evaluations showed that all documented species are effective in the treatment of cancer especially *Syzygium aqueum*, *Syzygium aromaticum*, *Syzygium cumini*, and *Syzygium samarangense* are rich sources of phytochemical constituents. It is proved that *Syzygium* genus is a source of bioactivity in the Myrtaceae family. We can infer from this systematic review that members of the genus *Syzygium* preferentially suppress the proliferation of all types of cancer cells tested and has no effect on normal cells. The systematic review, therefore, recommends further studies in the following species in terms of clinical trials, effective dosage, toxicity, and actual mechanism of action of the plant extract or the isolated compounds. Drug development and discovery from the genus *Syzygium* necessitates multidisciplinary scientific study.

## Abbreviations

ARE: antioxidant response element
*Bax*: B-cell lymphoma 2-associated X protein
*Bcl-2*: B-cell lymphoma 2
BSLT: brine shrimp lethality test
EAC: Ehrlich ascites carcinoma
IC_50_: half maximal inhibitory concentration
LC_50_: median lethal concentration
LD_50_: median lethal dose
MTT: 3-[4,5-dimethylthiazol-2-yl]2,5-diphenyl tetrazolium bromide
ROS: reactive oxygen species
SAEO: *Syzygium aromaticum* essential oil

## Declarations

### Copyright

© The Author(s) 2023.
